# BMC Complementary and Alternative Medicine reviewer acknowledgement 2012

**DOI:** 10.1186/1472-6882-13-57

**Published:** 2013-03-22

**Authors:** Thomas A Rowles

**Affiliations:** 1 BioMed Central, Floor 6, 236 Gray's Inn Road, London, WC1X 8HB, United Kingdom

## Abstract

**Contributing reviewers:**

The editors of BMC Complementary and Alternative Medicine would like to thank all our reviewers who have contributed to the journal in Volume 12 (2012).

## 

Samuel Abariga

USA

Muzaffar Abbas

USA

Hanaa Abd El Baky

Egypt

Omar Abdel Salam

Egypt

Azza Abdel-Aty

Egypt

Shereen Abdelghaffar

Egypt

Amin Malik Shah Abdul Majid

Malaysia

Luiz Carlos De Abreu

Brazil

Adakole Abu

Nigeria

Rana Abu-Dahab

Jordan

Vera Adamkova

Czech Republic

Adejuwon Adeneye

Nigeria

Sunday Adesegun

Nigeria

Achyut Adhikari

Pakistan

Amegnona Agbonon

Togo

Gabriel Agbor

Cameroon

Bharat Aggarwal

USA

María Graciela Agüero

Argentina

Joseph K Agyin

USA

Bashir Ahmad

Pakistan

Dilshad Ahmad

Saudi Arabia

Faiyaz Ahmed

India

Khaled Ahmed

Malaysia

Kwang Seok Ahn

South Korea

Jing Ai

China

Mikel Aickin

USA

Abdalrahim Aisha

Malaysia

Slimane Ait-Si-Ali

France

T.A. Ajith

India

Yoshihiko Akakabe

Japan

Murat Aksoy

Turkey

Syed Imteyaz Alam

India

Mohammad Sarwar Alam

India

Francisco Javier Alarcon-Aguilar

Mexico

Niaz Ali

Pakistan

Mauro Alini

Switzerland

Noorjahan Alitheen

Malaysia

Karim Alkadhi

USA

Kelli Allen

USA

Jackson Almeida

Brazil

Terje Alraek

Norway

Ragaa Hosny Mohamad Al-Tahtawy

Egypt

Cosme Alvarado-Esquivel

Mexico

Adel Al-Zubairi

Saudi Arabia

George Jimboyeka Amabeoku

South Africa

Massoud Amanlou

Iran

Ryszard Amarowicz

Poland

Omar Ameer

Australia

Saeed Amel Jamedar

Iran

Zahra Amin

Malaysia

Kamal Amin

Egypt

Marie Josephe Amiot

France

Hyojin An

South Korea

Arturo Anadon

Spain

Gerhard Andersson

Sweden

Ruby Anto

India

Nuno Antunes

USA

Kingsley Anukam

Nigeria

Chippada Appo

India

Natalia Aptsiauri

Spain

Farrukh Aqil

USA

Mohammed Aqil

India

Danielle Aragão

Brazil

Makoto Arai

Japan

Adeyemi Oladapo Aremu

South Africa

Maurice Arnaud

Switzerland

Julia T. Arnold

USA

Manuel Arroyo-Morales

Spain

Kristen Arthur

USA

Majid Artus

United Kingdom

Akalpita Arvindekar

India

George Asare

Ghana

Hossam Ashour

Egypt

Rehab Ashour

Egypt

Jules Clement Nguedia Assob

Cameroon

Akram Astani

Iran

John Astin

USA

Doina Atanasiu

USA

Bharathi Avula

USA

Iben Axén

Sweden

Itai Bab

Israel

Koichi Baba

Japan

Horacio Bach

Canada

Hyunsu Bae

South Korea

Victor Bagla

South Africa

Vivek K. Bajpai

South Korea

Manjeshwar Shrinath Baliga

India

Lynda Balneaves

Canada

Ju Yeon Ban

South Korea

Vassya Bankova

Bulgaria

Jameela Banu

USA

Paula Baptista

Portugal

Lu&Iacute;S Barbisan

Brazil

Stefania-Felicia Barbuceanu

Romania

Matthew Philip Greig Barnett

New Zealand

Gil Bar-Sela

Israel

Ronald Hma Bartels

Netherlands

Subal C Basak

India

K. Husnu Can Baser

Turkey

Maurizio Battino

Italy

Brent Bauer

USA

Patrick Bednarski

Germany

Shahnur Begum

Malaysia

Iris Bell

USA

Alexandre Bella Cruz

Brazil

Paolo Bellavite

Italy

Hedi Ben Mansour

Tunisia

Eran Ben-Arye

Israel

Obdulio Benavente-García

Spain

Elizaveta Benevolenskaya

USA

Alan Bensoussan

Australia

Alka Beotra

India

Luciano Bernardi

Italy

Joanne Bero

Belgium

Smrati Bhadauria

India

Srinivas Bharath

India

Soumya Bhattacharyya

India

Wissem Bhouri

Tunisia

Zhaoxiang Bian

Hong Kong

Zhongqi Bian

China

Zhuan Bian

China

Xavier Bigard

France

Papiya Bigoniya

India

Jennifer Billinsky

Canada

Gurjeet Birdee

USA

Felicity Bishop

United Kingdom

Li Bo

China

Virgilio Bocanegra-Garcia

Mexico

Katja Boehm

Germany

Kath Bogie

USA

Aline Boligon

Brazil

Heather Boon

Canada

Patcharee Boonsiri

Thailand

Mohammad Hossein Boskabady

Iran

Marco Botelho

Brazil

Francien Botha

South Africa

Ines Bouhlel

France

H Leon Bradlow

USA

Alex Braiman

Israel

Giorgio Brandi

Italy

Lesley Braun

Australia

Maurizio Brizzi

Italy

Caragh Brosnan

Australia

Iain Brownlee

United Kingdom

Edyta Brzoska

Poland

Dorly Buchi

Brazil

Arndt Buessing

Germany

Rainer W Bussmann

USA

Monica Butnariu

Romania

Carlo Calabrese

USA

Gioacchino Calapai

Italy

Candelario Calibo

Philippines

Marcus Calkins

USA

Melainie Cameron

Australia

Bruce Campbell

USA

Maria Martha Campos

Brazil

Margarita Canales

Mexico

Wangsen Cao

USA

Huijuan Cao

China

Boyang Cao

China

Peng Cao

China

Denise Carmona Cara

Brazil

Maria Carpinella

Argentina

Miguel Carro-Juárez

Mexico

Brenda Cartmel

USA

Marcelo Carvalho

Brazil

Adelaida María Castro-Sánchez

Spain

Hugo Cerecetto

Uruguay

Laima Česonienė

Lithuania

Han-Jung Chae

South Korea

Kin Chak

Taiwan

Gopal Chakrabarti

India

Siva Challa

India

Sue Hay Chan

Malaysia

Chanpen Chanchao

Thailand

Sumitra Chanda

India

Sudhir Chandna

India

Yuan Shiun Chang

Taiwan

Bei-Hung Chang

USA

Fang-Rong Chang

Taiwan

Shang-Tzen Chang

Taiwan

Jane Chao

Taiwan

Debprasad Chattopadhyay

India

Nagendra Chauhan

India

Haw-Wen Chen

Taiwan

Kevin Chen

USA

Rui Chen

China

Chao-Yin Chen

USA

Shih-Shun Chen

Taiwan

Guoxun Chen

USA

Pau-Chung Chen

Taiwan

Jin-Jer Chen

Taiwan

Nien-Jung Chen

Taiwan

Yi-Hung Chen

Taiwan

Wanshen Chen

China

Kuei-Min Chen

Taiwan

Yuh-Lien Chen

Taiwan

Si Chen

USA

Shilin Chen

China

Wen-Chi Chen

Taiwan

Yung-Hsiang Chen

Taiwan

Ling Cheng

USA

Evan Cherniack

USA

Daniel Cheuk

Hong Kong

Corjena Cheung

USA

Chin-Wen Chi

Taiwan

Chih-Yen Chien

Taiwan

Tinatin Chikovani

Georgia

Zahid Chohan

Pakistan

Byung Tae Choi

South Korea

Hye Sun Choi

South Korea

Kowityu Chong

Taiwan

Ke Dan Chu

China

Tsung-Hsien Chuang

Taiwan

Jude Chukwujekwu

South Africa

Kwang Chul Chung

South Korea

Vincent Chung

China

Yong-An Chung

South Korea

Sasitorn Chusri

Thailand

Chih-Pin Chuu

Taiwan

Richard Cimanga Kanyanga

Belgium

Haci Ibrahim Cimen

Turkey

Matthias Clauss

USA

Yuri Clement

Trinidad and Tobago

Ahmet Yilmaz Coban

Turkey

Rodney Colina

Uruguay

Michael Colvard

USA

Werner Cordier

South Africa

Steyner Côrtes

Brazil

Marco Cosentino

Italy

Cindy Crawford

USA

Diane Cridennda

USA

Francisco Cruz-Sosa

Mexico

Dezso Csupor

Hungary

Roberto Cuman

Brazil

Douglas Curran-Everett

USA

Sarada D.V.L.

India

José Eduardo Da Silva-Santos

Brazil

Konstantinos Dabos

Greece

Zhijun Dai

China

Eduardo Dalmarco

Brazil

Rula M. Darwish

Jordan

Jacques Y Datte

Cote d'Ivoire

Jonathan Davidson

USA

De An De An Guo

China

Laura De Martino

Italy

Damião De Sousa

Brazil

Helene De Wet

South Africa

Afef Dellai

Yemen

Changqing Deng

China

Xuming Deng

China

Krupali Desai

USA

Michael Deschenes

USA

Miranda Deutschlander

South Africa

Ranjitsinh Devkar

India

Saikat Dewanjee

India

Sanjit Dey

India

Suranganie Dharmawardhane

USA

Vermont P Dia

USA

Phil Dinning

Australia

Prakash Diwan

India

Preeti Dohare

USA

Lianne Dolan

Canada

Xin Du

China

Nawal Dubey

India

Shailesh Dudhgaonkar

India

Aurelien Dumetre

France

Veeramuthu Duraipandiyan

India

Jeffery Dusek

USA

Rafael Cypriano Dutra

Brazil

B.S. Dwarakanath

India

Chandradhar Dwivedi

USA

Jean Paul Dzoyem

Cameroon

Ye Earm

South Korea

Mohammad Ali Ebrahimzadeh

Iran

Ronald Eccles

United Kingdom

Tamer Edirne

Turkey

Valerie Edwards-Jones

United Kingdom

Thomas Efferth

Germany

Yusuke Egashira

Japan

Tolga Eichhorn

Germany

Linda Einbond

USA

Stephen Ekunwe

USA

Mahmoud El Kouni,

USA

Hesham El-Beshbishy

Saudi Arabia

Siham Elshenawy

Egypt

Walid El-Tantawy

Egypt

Asongalem Emmanuel Acha

Cameroon

Ephrem Engidawork

Ethiopia

Hui Meng Er

Malaysia

Gokhan Eraslan

Turkey

Edzard Ernst

United Kingdom

Wichai Eungpinichpong

Thailand

Marion Willard Evans Jr

USA

Caroline Eyles

United Kingdom

Titilayo Fakeye

Nigeria

Yi-Ming Fan

China

Jianglin Fan

Japan

Evandro Fang

USA

Jianqiao Fang

China

Murad Faris

USA

Per G Farup

Norway

Elizaveta Fasler-Kan

Switzerland

Sadaf Fatima

India

Olaniyi Fawole

South Africa

Raja Fayad

USA

Richelle Jeannette Fyline Felt

Netherlands

Gustavo Ferreira

Brazil

T Field

USA

Branko Filipović

Serbia

Susana Fiorentino

Colombia

Peter Fisher

United Kingdom

Margaret Fitch

Canada

Leon Flicker

Australia

Swaran Flora

India

Vinjar Fonnebo

Norway

Romain Forestier

France

Gail Fraizer

USA

David Freier

USA

Yolanda Freile

Mexico

Rivelilson Freitas

Brazil

Carmelita Frondoza

USA

Liang-Wu Fu

USA

Hidefumi Fukumitsu

Japan

János Gaál

Hungary

Chhaya Gadgoli

India

Hala Gali-Muhtasib

Lebanon

Moreno Galleni

Belgium

Sylvia Galloway

USA

Julio Galvez

Spain

Hin Hark Gan

USA

Jianfei Gao

China

Virginia Garcia

Spain

Gerd Gauglitz

Germany

Charly Gaul

Germany

Stephen Gbedema

Ghana

Edyta Gendaszewska-Darmach

Poland

Patricia Gerbarg

USA

Lee Geun-Soo

South Korea

Saroj Ghaskadbi

India

M. Nabeel Ghayur

Canada

Osama Gheith

Egypt

Anwar Gilani

Pakistan

Andrea Giusti

Italy

Eiichi Gohda

Japan

Mohammad Jafar Golalipour

Iran

Michael Goldstein

USA

Andreia Gomes

Portugal

Yuewen Gong

Canada

Mercedes Gonzalez

Uruguay

Ma. Eva Gonzalez-Trujano

Mexico

Velliyur Kanniappan Gopalakrishnan

India

Joyce Govinden Soulange

Mauritius

Suzanne Grant

Australia

Sara Greay

Australia

Frank Greenway

USA

Jeffrey Greeson

USA

Sameline Grimsgaard

Norway

Gloria Gronowicz

USA

Enzo Grossi

Italy

Oliver Grundmann

USA

Yihui Guan

China

Kavita Gulati

India

Ilhami Gulcin

Turkey

Muhammad Gulfraz

Pakistan

Zhi-Ling Guo

USA

Praveena Gupta

USA

Subash Gupta

USA

Mitchell Haas

USA

Julie Hadley

United Kingdom

Hani Hafez

Saudi Arabia

Akihiro Hagiwara

Japan

Valiollah Hajhashemi

Iran

Mannan Hajimahmoodi

Iran

Ahmad Sukari Halim

Malaysia

Helen Hall

Australia

Richard Hammerschlag

USA

Alaaeldin Hamza

United Arab Emirates

Ji-Sheng Han

China

Alex Hankey

India

Huang Hann-Jang

Taiwan

John Hanover

USA

Sheng-Po Hao

Taiwan

Hideaki Hara

Japan

Hirokazu Hara

Japan

Kaleeckal Harikumar

USA

Abhay Harsulkar

India

Syed Shahzad Hasan

Malaysia

Tarique Hasan

India

Tahereh Hasanloo

Iran

Lahcen Hassani

Morocco

Noboru Hattori

Japan

Apostolos Hatzitolios

Greece

Li He

China

Xiaojuan He

China

David Heber

USA

Markus Helfer

Germany

Ralf Henkel

South Africa

Paul Henschke

Australia

Patricia Herman

USA

Maribel Herrera Ruiz

Mexico

Hugo Hesser

Sweden

John Highton

New Zealand

Susan Hillier

Australia

Brian Hinote

USA

Masayuki Hiramatsu

Japan

Tadashi Hisamitsu

Japan

Emily Ho

USA

Kathleen Hoeger

USA

Bastian Hoesel

Austria

Robert Hoffman

USA

Michael Höfler

Germany

Judit Hohmann

Hungary

Lone Holst

Norway

Kam-Lun Ellis Hon

Hong Kong

Saeyong Hong

South Korea

Shirin Hooshmand

USA

Katarina Hostanska

Switzerland

Wen-Chi Hou

Taiwan

Ching-Liang Hsieh

Taiwan

Hsi-Lung Hsieh

Taiwan

Chin-Lin Hsu

Taiwan

Chien-Ming Hu

Taiwan

Bing Hu

China

Wei-Jan Huang

Taiwan

Xi Huang

China

Wendong Huang

USA

Tur-Fu Huang

Taiwan

Chi-Chang Huang

Taiwan

Donfack Hubert

Cameroon

Ruben Hummelen

Netherlands

Chi-Feng Hung

Taiwan

Kent Hunter

USA

Kazim Husain

Puerto Rico

Kazim Husain

USA

Khalid Hussain

Pakistan

Ivo Iavicoli

Italy

Etinosa Igbinosa

Nigeria

Savarimuthu Ignacimuthu

India

John Igoli

Nigeria

Sin-Hyeog Im

South Korea

Lionel In

Malaysia

Naoki Inagaki

Japan

Osamu Inanami

Japan

Kouno Isao

Japan

Torao Ishida

Japan

Marina Isidori

Italy

Eric Jacobson

USA

Ganesh Chandra Jagetia

India

Umesh K Jain

India

Erica James

Australia

Polona Jamnik

Slovenia

Tong-Rong Jan

Taiwan

Choon-Gon Jang

South Korea

Insoo Jang

South Korea

Stanislav Janousek

Czech Republic

Nattinee Jantaratnotai

Canada

Zenon Jastrzebski

Poland

Hye Gwang Jeong

South Korea

Yongri Jin

USA

Chen Jing

China

Jiraphun Jittikoon

Thailand

Karin Joehrer

Austria

Jeremy Johnson

USA

Miek Jong

Netherlands

Myungsoo Joo

South Korea

Stefanie Joos

Germany

Scott Jordan

Canada

Joerg Jores

Kenya

Elizabeth Joseph

USA

Wan Jy

China

Kiran Kalia

India

Kazuki Kanazawa

Japan

Masayuki Kaneko

Japan

Izuru Kaneko

Japan

Hee Kang

South Korea

Suman Kapila

India

Sherif Karam

United Arab Emirates

Devarajan Karunagaran

India

Violet Kasabri

Jordan

Rajpal Kashyap

India

Candida Kassuya

Brazil

Benjamin Katchman

USA

Manjinder Kaur

USA

Duygu Kaya

Turkey

Idowu Kazeem

Nigeria

Paul Kelly

United Kingdom

Kathi Kemper

USA

Noha Khalaf

Egypt

Saeed Khan

USA

Naveed Khan

Pakistan

Fariba Khodagholi

Iran

Ar Khuda-Bukhsh

India

Sung Hwan Ki

South Korea

Jong-Choon Kim

South Korea

Song-Yi Kim

South Korea

Mi-Yeon Kim

South Korea

Seong Hwan Kim

South Korea

Jin-Wook Kim

South Korea

Sung Kim

South Korea

Chang-Ju Kim

South Korea

Mee Ree Kim

South Korea

Hollis King

USA

Kathryn King

Canada

Karl Kingsley

USA

Emma Kirby

Australia

Shiroh Kishioka

Japan

Takio Kitazawa

Japan

Marlise Klein

USA

Benjamin Kligler

USA

Uwe Knippschild

Germany

Seong-Gyu Ko

South Korea

Alexander Koch

Germany

Akiko Kojima-Yuasa

Japan

Badireenath Konkimalla

India

Malcolm Koo

Canada

Muberra Kosar

Turkey

Demetrios Kouretas

Greece

Sharon Wald Krauss

USA

Nandakumar Krishnadas

India

Nateetip Krishnamra

Thailand

Georg Krupitza

Austria

Sae Kwang Ku

South Korea

Jules-Roger Kuiate

Cameroon

Malgorzata Kujawska

Poland

Takashi Kumagai

Japan

Vijaya Kumar

India

Pardeep Kumar

India

Chidambaram Kumarappan

Malaysia

Ajaikumar Kunnumakkara

USA

Amit Kunwar

India

Ichiro Kurokawa

Japan

Masahiko Kurokawa

Japan

Dong-Yeul Kwon

South Korea

Hokeun Kwon

USA

Youngjoo Kwon

South Korea

Joao Lago

Brazil

Helene Langevin

USA

Malinee Laopaiboon

Thailand

Olga Leaños

Mexico

Myeong Soo Lee

South Korea

Kun-Ta Lee

Taiwan

Min Won Lee

South Korea

Young-Cheol Lee

South Korea

Yun Jong Lee

South Korea

Tat-Leang Lee

Singapore

Eui Ju Lee

South Korea

Kyoung-Youl Lee

South Korea

Siwoo Lee

South Korea

Jang-Hern Lee

South Korea

David Lee

USA

Eunah Lee

South Korea

Choong-Gu Lee

South Korea

Yong Heum Lee

South Korea

Anne Leis

Canada

Joseph C K Leung

Hong Kong

George Lewith

United Kingdom

Peng Li

USA

Xican Li

China

Min Li

USA

Shao Li

China

Zhiyong Li

China

Jian-Xin Li

China

Aihui Li

USA

Ping Li

China

Gong Liang

China

Liunn-Wang Liao

Taiwan

Edgars Liepins

Latvia

Wan-Wan Lin

Taiwan

Jung-Yaw Lin

Taiwan

Yaw-Huei Lin

Taiwan

Bi-Fong Lin

Taiwan

Hongsheng Lin

China

Cheng-Wen Lin

Taiwan

Hsueh-Kung Lin

USA

Wen-Chuan Lin

Taiwan

Klaus Linde

Germany

Ulrike Lindequist

Germany

Robert Listecki

USA

Jianping Liu

China

Jia-Jun Liu

China

Chan-Min Liu

China

Yitong Liu

USA

Ping Liu

China

Audhild Lohre

Norway

Yijia Lou

Comoros

Jun Lu

New Zealand

Waldecy Lucca Jr

Brazil

Chi-Wai Lui

Australia

Anderson Luiz-Ferreira

Brazil

J Lydia

India

Zhi-Zhong Ma

China

Daqing Ma

United Kingdom

Ning Ma

Japan

Danielle Macedo

Brazil

Bharti Magazine

India

Gail Mahady

USA

Jamal Mahajna

Israel

Mahassine Mahassine

Morocco

Messarah Mahfoud

Algeria

Emanuela Maioli

Italy

Runner R. T. Majinda

Botswana

Juraj Majtan

Slovakia

Suzana Makpol

Malaysia

Hassan Malekinejad

Netherlands

Russell Malmberg

USA

Mao-Qiang Man

USA

Thamilvaani Manaharan

Malaysia

Nicasio Mancini

Italy

Nripendranath Mandal

India

Sanat Mandal

Canada

Prasenjit Manna

India

Nedeljko Manojlovic

Serbia

Lucia Manso-Silvan

France

Carla Marchetti

Italy

Murilo Marchioro

Brazil

Sheila Maregesi

Tanzania

Manuel Martinez Garcia

Spain

Stefania Marzocco

Italy

Khalid Matalka

Jordan

Robert Mathie

United Kingdom

Rajani Mathur

India

Isao Matsui-Yuasa

Japan

Hiroshi Matsushita

Japan

Harald Matthes

Germany

Ojezele Matthew

Nigeria

Brian Matthews

Australia

Jean Emmanuel Mbosso Teinkela

Cameroon

Florence Mbuh

USA

Pauline Mccabe

Australia

Malcolm Mccoubrie

United Kingdom

Lyndy Mcgaw

South Africa

Deirdre Mclaughlin

Australia

Philip Mcternan

United Kingdom

Isac Medeiros

Brazil

Ashish Mehta

Singapore

Darshan Mehta

USA

Rajendra Mehta

USA

Martina Meinke

Germany

Sandra Mendoza

Mexico

Nizar Mhaidat

Jordan

Shi-Chuen Miaw

Taiwan

Andreas Michalsen

Germany

Maria Leticia Miranda Fernandes Estevinho

Portugal

Shiraz Mishra

USA

Scott Mist

USA

Megha Mittal

India

Kazuo Miyashita

Japan

Ali Mobasheri

United Kingdom

Tarek Mohamed

Egypt

Fereidoon Mojtahed Jaberi

Iran

Abu-Asab Mones

USA

Ali Montazeri

Iran

Giuseppe Morgia

Italy

Mainen Moshi

Tanzania

Ali Mostafaie

Iran

Mahbub Mostofa

Bangladesh

Yoshiharu Motoo

Japan

Kamal Moudgil

USA

Mack Moyo

South Africa

Ruiling Mu

China

Ruiling Mu

USA

Alister Muir

Canada

Cephas Tagumirwa Musabayane

South Africa

Novatus Mushi

Tanzania

Mohd Rais Mustafa

Malaysia

Mustofa Mustofa

Indonesia

Ramanathan Muthiah

India

Kesara Na-Bangchang

Thailand

Seyed Mohammad Nabavi

Iran

H R Nagendra

India

Richard Nahin

USA

Hossein Nahrevanian

Iran

Vinny Naidoo

South Africa

Suresh Naik

India

Hareesh Nair

USA

Wadie Najm

USA

Yuichiro Nakada

Japan

Toshiya Nakamura

Japan

Gopalan Kutty Nampurath

India

Mark Nanyingi

Kenya

Marjan Nassiri-Asl

Iran

Declan Naughton

United Kingdom

Yogendra Nayak

India

Shivananda Nayak

Trinidad and Tobago

Veena Nayak

India

Ashwell Ndhlala

South Africa

Vasanthakumari Neela

Malaysia

Pratibha Nerurkar

USA

Telesphore Benoit Nguelefack

Cameroon

J. David N'Guessan

Cote d'Ivoire

Glenda Nicioli Da Silva

Brazil

Sunil Nirmal

India

Yuhei Nishimura

Japan

Jessica Noggle

USA

Emeka Nweze

Nigeria

Stephen Samwel Nyandoro

Tanzania

Ivan Nyklicek

Netherlands

Ganiyu Oboh

Nigeria

Kylie O'Brien

Australia

Daniel O'Connor

Australia

You-Chang Oh

South Korea

Byeongsang Oh

Australia

Osamu Ohno

Japan

Roberta Okamoto

Brazil

Charles Okoli

Nigeria

Stephen Okpo

Nigeria

Kensuke Okuda

Japan

Nada Orsolic

Croatia

Iwao Osaka

Japan

Mike Yaw Osei-Atweneboana

Ghana

Kazeem Oshikoya

Nigeria

Thomas Ostermann

Germany

Giacomo Oteri

Italy

Hsiu-Chung Ou

Taiwan

Romy Ouziel

Belgium

Ashli Owen-Smith

USA

Bamidele Owoyele

Nigeria

Vidhu Pachauri

India

Andreas Pahl

Germany

Sreedhara Ranganath Pai

India

Dasja Pajkrt

Netherlands

Arttatrana Pal

India

'Afa Palu

USA

Min-Hsiung Pan

Taiwan

John Pan

USA

Tai-Long Pan

Taiwan

Anurag Pandey

India

Jong-Hwei Pang

Taiwan

Pharkphoom Panichayupakaranant

Thailand

Ampai Panthong

Thailand

Leila Parente

Brazil

Ji-Eun Park

South Korea

Jeong Hyun Park

South Korea

Hi-Joon Park

South Korea

Ignacio Parraga

Spain

Jagan Mohan Patlolla

USA

Bhushan Patwardhan

India

Christina Gundgaard Pedersen

Denmark

Karl Peltzer

South Africa

Jose Peña

Spain

Wen-Huang Peng

Taiwan

Guangyong Peng

USA

Jun Peng

China

David Pereira

Portugal

Patricia Pereira

Portugal

Rosa M Perez-Gutierrez

Mexico

Duong Duc Pham

South Korea

Preecha Phuwapraisirisan

Thailand

Watcho Pierre

Cameroon

Andrzej Pilc

Poland

Karen Pilkington

United Kingdom

Somchai Pinlaor

Thailand

Marie Pirotta

Australia

Frederico Pittella

Japan

Beth Poindexter

USA

Moschos Polissiou

Greece

Siddhartha Popat

Germany

Ada Popolo

Italy

Seppan Prakash

India

K. Prasad

Malaysia

Balakrishnan Prithiviraj

Canada

Maulik Purohit

USA

Xianjun Qu

China

Hude Quan

Canada

Ana Quesada

Spain

Satyan R S

India

Atiar Rahman

Bangladesh

Asmah Rahmat

Malaysia

Mahendra Rai

India

Komal Raina

USA

Sudhakar Raja

India

Aiyalu Rajasekaran

India

Yallappa Rajashekar

India

Ilavarasan Raju

India

Kalavathy Ramasamy

Malaysia

Branislav Rankovic

Serbia

Elia Ranzato

Italy

Shaival Kamalaksha Rao

India

Azhar Rasul

China

Jo-Anne Rayner

Australia

Vivienne Reeve

Australia

Leonardo Reis

Brazil

Clare Relton

United Kingdom

Gülin Renda

Turkey

Flavia Aparecida Resende

Brazil

Wanda Reygaert

USA

Abirami Rg

India

Man Hee Rhee

South Korea

Marit By Rise

Norway

Praveen Rishi

India

Maria Dolores Rivero-Perez

Spain

Ramon Enrique Robles Zepeda

Mexico

Gabriela Rodriguez-Manzo

Federated States of Micronesia

Eugene Rogozhin

Russian Federation

Janne Rojas

Venezuela

Maan Bahadur Rokaya

Czech Republic

Tovit Rosenzweig

Israel

Bishnupada Roy

India

Eduardo Ruiz-Bustos

Mexico

Rod Russell

Canada

Lex Rutten

Netherlands

Sandhya S

India

Niina Saarinen

Finland

Ali Sabahi

USA

Suna Sabuncuoglu

Belgium

Richard Sadovsky

USA

Dinkar Sahal

India

Umadevi Sajjan

USA

Takako Sakamoto

Japan

Naoki Sakane

Japan

Luis Salazar-Olivo

Mexico

Noah Samuels

Israel

Christelle Sanchez

Belgium

Sara Serafina Sánchez

Argentina

Louis Pergaud Sandjo

Cameroon

Sankar Sanyal

India

Lucky Saraswat

United Kingdom

Kumar A Saravana

India

Jerome Sarris

Australia

Sreenivasan Sasidharan

Malaysia

Tadaaki Satou

Japan

Ravindra Satpute

India

Alexander Schauss

USA

Manfred Schedlowski

Germany

Christian Scheffer

Germany

Valerie Schini-Kerth

France

Patricia Fernanda Schuck

Brazil

Zev Schuman-Olivier

USA

Elita Scio

Brazil

Anna Ivana Scovassi

Italy

Julie Secombe

USA

Dugald Seely

Canada

Teet Seene

Estonia

Shuichi Segawa

Japan

Susan Semple

Australia

Tuhinadri Sen

India

Jose Sforcin

Brazil

Maziar Shafighi

Switzerland

Hongcai Shang

China

Wenbin Shang

China

Manjula Shantaram

India

Joseph Shapiro

USA

Girish Sharma

India

Ajay Sharma

South Korea

Shyam Sharma

India

Gunjan Sharma

India

Ashoke Sharon

India

Xueyong Shen

China

Xiao Shen

Switzerland

Xiaoyan Shen

China

Rekha Shenoy

India

Smita Shenoy

United Kingdom

Su Shi-Bing

China

Yasutaka Shigemura

Japan

Chun-Ching Shih

Taiwan

Heung Shin

South Korea

Byung-Cheul Shin

South Korea

Dong-Hwa Shon

South Korea

Chengchao Shou

China

Shruti Shukla

India

Shruti Shukla

South Korea

Sina Siavash Moghaddam

Iran

Agnieszka Siejka

Poland

Victor Sierpina

USA

Bagnolia Silva

Brazil

Maree Simpson

Australia

Minni Singh

India

Sukh Mahendra Singh

India

Satendra Singh

South Africa

Divya Singh

India

Fuschia M. Sirois

Canada

Pongtip Sithisarn

Thailand

Helen Skaltsa

Greece

Dan Sliva

USA

Sandra Smith

USA

Caroline A Smith

Australia

Eduardo Sobarzo-Sánchez

Spain

Abayomi Sofowora

Nigeria

Didem Sohretoglu

Turkey

Slavica Solujic

Serbia

Rajan Somasundaram

Germany

Hwa-Young Son

South Korea

Chang Gue Son

South Korea

Sarangapani Sreelatha

Singapore

Bungorn Sripanidkulchai

Thailand

Eri Srivatsan

USA

Nicola Stagg

USA

Tatjana Stanojkovic

Serbia

Vanessa Steenkamp

South Africa

Pablo Steinberg

Germany

Aslak Steinsbekk

Norway

Audrey Stepien

USA

Joanna Stewart

New Zealand

Lily Stojanovska

Australia

Tracy Stuardi

USA

Barbara Stussman

USA

Xiulan Su

China

Chun-Li Su

Taiwan

Sorimuthu Subramanian

India

Thyagarajan Subramanian

USA

Nikolaus Sucher

USA

Lonchin Suguna

India

Gil Joon Suh

South Korea

Shaida Fariza Sulaiman

Malaysia

Mohd Roslan Sulaiman

Malaysia

Siti Amrah Sulaiman

Malaysia

Wei-Zen Sun

Taiwan

Ming-Yu Sun

China

Tobias Sundberg

Sweden

Roongtawan Supabphol

Thailand

Martina Sutovska

Slovakia

Masumi Suzui

Japan

Akira Suzuki

Japan

Thippeswamy Swamy

India

Naveed Ahmed Syed

India

Juan Sztajzel

Switzerland

Agnieszka Szuster

Poland

Toku Takahashi

USA

Noburai Takakura

Japan

Hiroaki Takimoto

Japan

Jean De Dieu Tamokou

Cameroon

Hiroomi Tamura

Japan

Hiroyuki Tanaka

Japan

Chanderdeep Tandon

India

Pierre Tane

Cameroon

Beisha Tang

China

Wei Tang

Japan

Yaxiong Tang

China

Alejandro Tapia

Argentina

Rosamaria Tedeschi

Italy

Gerald Ngo Teke

Cameroon

Rajeshwar Rao Tekmal

USA

Nitin Telang

USA

Shirley Telles

India

Rémy Bertrand Teponno

Cameroon

Luisa Tesoriere

Italy

Sarada Tetali

India

Michael Teut

Germany

Mayank Thakur

India

Arno Thaller

Germany

Kavitha Thirumurugan

India

Tamsyn Thring

United Kingdom

Jie Tian

China

Evelin Tiralongo

Australia

Stephanie Tjen-A-Looi

USA

Michal Toborek

USA

Selma Tobudic

Austria

Ariel Toloza

Argentina

Mark Tonelli

USA

Masaru Toriyama

Japan

Fátima Torrico

Venezuela

Shalini Tripathi

India

Hector W.H. Tsang

Hong Kong

Karl Wk Tsim

China

Takanori Tsuda

Japan

Kazuhiro Tsuruma

Japan

Salma Tukan

Jordan

Siriporn Tuntipopipat

Thailand

Mustafa Turan

Turkey

Gaffari Turk

Turkey

Yew-Min Tzeng

Taiwan

Shaikh Uddin

Australia

Sigurd Uldall

Denmark

Ikram Ullah

South Korea

Dawn Upchurch

USA

Padmaja Vaidyanathan

India

Gustavo Valencia Del Toro

Mexico

Eva Van Der Ploeg

Australia

Leo Van Griensven

Netherlands

An M.T. Van Nuffel

Belgium

Meena Vangalapati

India

Rajeev Varshney

India

Maria Helena Vasconcelos

Portugal

Vitor Vasconcelos

Portugal

Emmelyne Vasse

Netherlands

Mani Vasudevan

India

Marta Vattuone

Argentina

Michael Velarde

USA

Judith Velasco

Venezuela

Vaneja Velenik

Slovenia

Gopalakrishnan Velliyur Kanniappan

India

Hilde Verbeek

Netherlands

Claudio Viegas Jr

Brazil

Viswanadha Vijaya Padma

India

Fabiana Vilela

Brazil

Gines Viscor

Spain

Patricia Vit

Venezuela

Günter Vollmer

Germany

Gustavo Volpato

Brazil

Shreya Vora

India

Cyrus Githaiga Wagate

Kenya

Noemi Waksman

Mexico

Jianwei Wang

China

Qingguo Wang

China

Gang Wang

China

Ping Wang

USA

Bor-Sen Wang

Taiwan

Guan-Lei Wang

China

Ling Wang

China

Qi Wang

China

Jian Wang

USA

Xijun Wang

China

Diane Wardell

USA

Tim Watson

United Kingdom

Peter Wayne

USA

Connie Weaver

USA

Laura Weeks

Canada

Natthida Weerapreeyakul

Thailand

Wolfgang Weidenhammer

Germany

Johannes Westendorf

Germany

Jeffrey White

USA

Gustav Wik

Norway

Lisa Wilhelm

USA

Karen Willis

Australia

Michael Wink

Germany

Wipawee Winuthayanon

USA

Alice Wong

Hong Kong

Eric Woode

Ghana

Jackie Wootton

USA

Darong Wu

China

Chi-Rei Wu

Taiwan

Chieh-Hsi Wu

Taiwan

Chunfu Wu

China

Huan-Gan Wu

China

Li-Dong Wu

China

Christine Wu

USAR

Juraithip Wungsintaweekul

Thailand

Gwen Wyatt

USA

Lei Xi

USA

Qiang-Min Xie

China

Hao Xu

China

Zheng-Hong Xu

China

Zhihong Xue

China

Nik Soriani Yaacob

Malaysia

Vijayshree Yadav

USA

Abhishek Yadav

India

Funda Nuray Yalcin

Turkey

Kazuo Yamada

Japan

Haruki Yamada

Japan

Kohji Yamaki

Japan

Xianzhong Yan

China

Rui-Hua Yang

China

Siyoung Yang

South Korea

Zhen Yang

USA

Changqing Yang

China

Suh-Ching Yang

Taiwan

Qifeng Yang

China

Woong Mo Yang

South Korea

Chao-Hsun Yang

Taiwan

Amalia Yanni

Greece

Gloria Yeh

USA

Ching-Hua Yeh

Taiwan

Chia Jui Yen

Taiwan

Wing-Fai Yeung

Hong Kong

Ling-Huei Yih

Taiwan

Da-Hai Yu

USA

Hua Yu

USA

Jong Won Yun

South Korea

Syed Faisal Zaidi

Japan

Shahrul Hisham Zainal Ariffin

Malaysia

Zainul Amiruddin Zakaria

Malaysia

Anna Zalewska-Janowska

Poland

Errol Zeiger

USA

Yanjun Zeng

China

Boli Zhang

China

Minzhou Zhang

China

Junzeng Zhang

Canada

Mingwei Zhang

China

Yan Zhang

USA

Tong Zhang

China

Xuan Zhang

USA

Zhang-Jin Zhang

Hong Kong

Anthony Zhang

Australia

Ying-Yong Zhao

China

Jun Zhao

China

Chao Zheng

USA

Guo-Qing Zheng

China

Xinyue Zhi

China

Yifei Zhong

China

Lei Zhou

USA

Wei Zhou

USA

Xuezhong Zhou

China

Xiaofeng Zhu

China

Yizhun Zhu

China

Muhammad Zia

Pakistan

Suzanna Zick

USA

Danuta Zielińska

Poland

Malek Zihlif

Jordan

Denis Zofou

Cameroon

Magdalena Zuk

Poland

Embong Zunaina

Malaysia

Zhenghong Zuo

China

## Pre-publication history

The pre-publication history for this paper can be accessed here:

http://www.biomedcentral.com/1472-6882/13/57/prepub

